# Lipoma Arborescens: Review of an Uncommon Cause for Swelling of the Knee

**DOI:** 10.1155/2016/9538075

**Published:** 2016-05-16

**Authors:** M. De Vleeschhouwer, E. Van Den Steen, G. Vanderstraeten, W. Huysse, J. De Neve, L. Vanden Bossche

**Affiliations:** ^1^Physical Medicine and Rehabilitation, Ghent University Hospital, 9000 Ghent, Belgium; ^2^Department of Physical Medicine and Rehabilitation, AZ Sint-Jan, 8000 Bruges, Belgium; ^3^Department of Radiology, Ghent University Hospital, 9000 Ghent, Belgium

## Abstract

Lipoma arborescens is a rare cause of chronic monoarticular arthritis, with only a few cases reported in the literature. It is most commonly seen in the knee, but cases in other joints such as the wrist, shoulder, and elbow have also been described. It is a benign condition, in which the subsynovial tissue is replaced diffusely by mature fat cells. We describe a case involving the knee and discuss the symptoms, diagnosis, and treatment.

## 1. Introduction

Lipoma arborescens, also referred to as villous lipomatous proliferation of synovial membrane, diffuse lipoma of the joint, or diffuse synovial lipoma, is a rare benign intra-articular lesion characterized by villous proliferation of the synovial membrane [1]. The etiology of this condition still remains unclear.

Lipoma arborescens typically affects adults. It most commonly involves the knee, but other locations have also been described. People present with joint pain, swelling, and effusion. The diagnosis is based on the typical appearance on MRI, and the recommended treatment is open or arthroscopic synovectomy. Recurrence is uncommon.

## 2. Case Report

A 31-year-old Dutch man of Turkish origin presented to the physical and rehabilitation medicine department with low back pain. On physical examination, a marked effusion in the right knee joint was noted. According to the patient, the swelling had been present since two years in a varying degree, with intermittent episodes of pain. There was no history of trauma. The patient's father had type-2 diabetes mellitus, but our patient was not diagnosed with it. The patient was known to have psoriasis.

**Figure 1:**
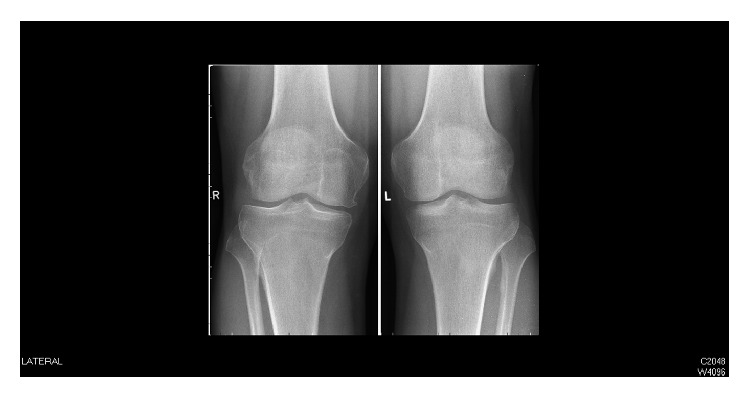
Radiography of the knees shows signs of arthrosis: a bony spur at the medial femoral condyle, femorotibial narrowing of the joint space, and a hydrops.

During the consultation, an aspiration was performed yielding 113 cc of citrine-colored fluid. The patient was prescribed rest and nonsteroidal anti-inflammatory drugs. Because the patient did not respond to this treatment, radiographs were obtained demonstrating effusion in the right knee and osteoarthritic changes (Figure 1).

**Figure 2:**
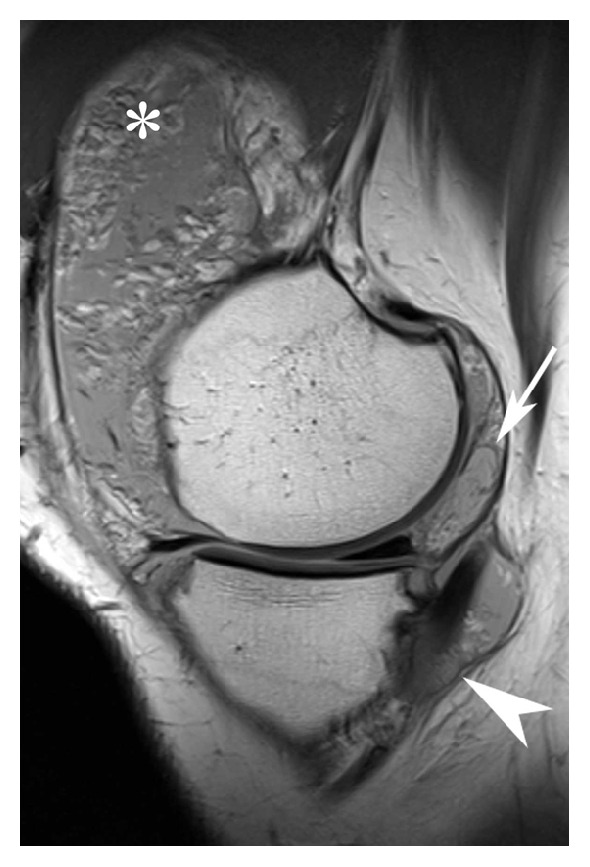
Sagittal proton-density weighted fast spin echo image through the medial femorotibial compartment showing not only a villous fatty proliferation of the synovial membrane in the suprapatellar recess (asterisk) but also a fatty mass posterior to the femoral condyle (arrow) and villous proliferations around the insertion of the semimembranosus tendon (arrowhead).

**Figure 3:**
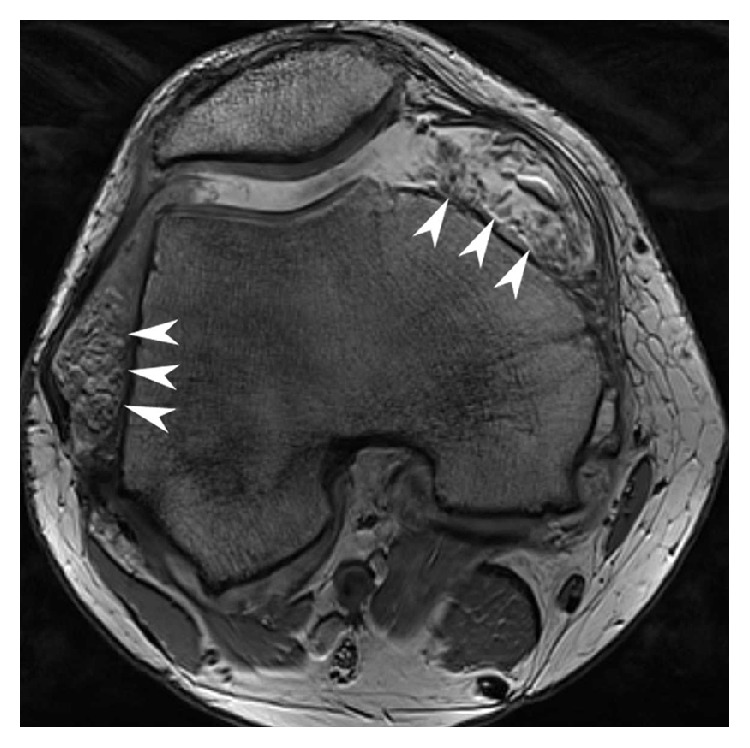
Transverse T2-weighted gradient echo (DESS 3D) image through the patellofemoral joint clearly depicts extensive synovial proliferations in the medial and lateral recess (arrowheads).

One week later, the patient again presented with recurrent swelling. An ultrasonographic examination disclosed a large amount of fluid in the suprapatellar recess, with synovial villi, but no hyperemia. A bony spur was seen on the medial femoral condyle. Laboratory tests showed no elevated inflammatory parameters, and negative rheumatoid factor, anti-CCP, and HLA-B27 typing. A bone scan combined with SPECT revealed mildly-to-moderately increased remodeling at both knees, but mainly on the right. MRI demonstrated moderate knee osteoarthritis, a region of chondropathy in the medial tibial cartilage, and marked effusion with multiple fatty synovial proliferations, which is pathognomonic of lipoma arborescens (Figures 2 and 3).

**Figure 4:**
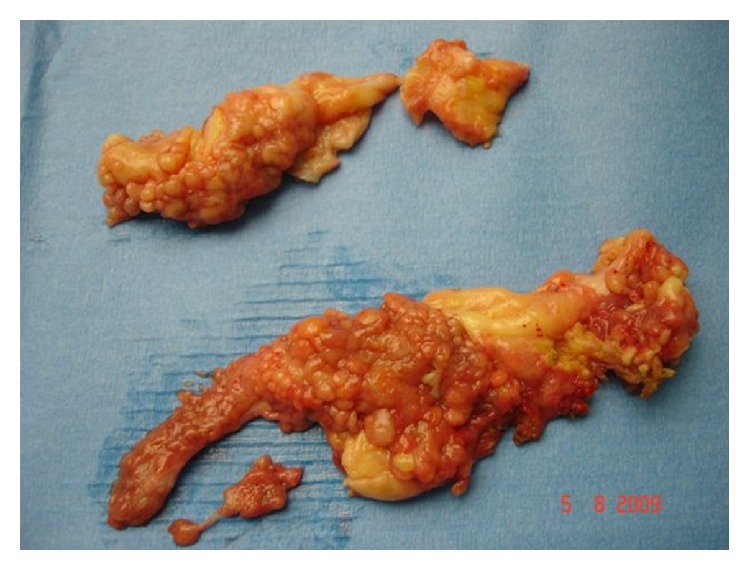
Lipoma arborescens after resection.

Six weeks later, an arthrotomy was performed with a total synovectomy and resection of hypertrophic multilobulated synovial tissue. The diagnosis of lipoma arborescens was confirmed on histological examination (Figure 4).

## 3. Discussion

Lipoma arborescens is a rare, mainly intra-articular lesion characterized by diffuse replacement of subsynovial tissue by mature fat cells, giving rise to a prominent villous transformation of the synovium [2]. It was first described by Hoffa in 1904, and more in detail in 1957 by Arzimanoglu [3, 4]. The term* lipoma* is misleading because the lesion does not show any macroscopic or histological features of a neoplasm. Therefore, Hallel et al. suggested that lipomatous proliferation of the synovial membrane would be a more appropriate name [2]. The term* arborescens *comes from the word arbor (Latin for tree) and describes the macroscopically treelike morphology of this lipomatous villous synovial proliferation [5].

Although the lesion is usually located within a joint, involvement of the subdeltoid and bicipital bursa [6] and the synovial sheath of the peroneal tendons around the ankle joint has also been described [7].

Lipoma arborescens usually affects the knee, preferentially the suprapatellar pouch, but has also been reported to occur in other joints such as the hip [5], wrist [8], elbow [9], and shoulder [10]. Although the condition is mainly unilateral, bilateral involvement and multiple joint involvement have also been described [5, 6, 11, 12].

Less than 100 cases of lipoma arborescens have been reported in the literature. The peak incidence is between the third and fifth decades, with a predilection for male [13]. There are only a few cases described in literature about children.

The etiology is still unknown, and although it can occur without antecedents, it is associated in the literature with trauma, inflammation, or inflammatory joint diseases, neoplasm, and degenerative conditions (osteoarthritis) [14, 15]. There was even one case report describing a relation to infection (septic arthritis) [16].

A synovial reaction triggered by a trauma has been postulated, but most patients with lipoma arborescens do not have a history of trauma and nor did our patient.

The hypothesis of lipoma arborescens being a reaction to chronic inflammation is supported by the histological finding of a mononuclear cell infiltrate in the underlying synovial membrane [13]. Furthermore, inflammatory conditions, such as rheumatoid arthritis, psoriatic arthritis, or psoriasis, uveitis, and juvenile spondyloarthropathy, are in several reports associated with lipoma arborescens [17, 18].

A causal relationship between lipoma arborescens and degenerative joint disease is suspected but has yet to be confirmed. It has been suggested that the lesion can be divided into a primary and a secondary type. The primary type is rare and has been described as a form of synovial lipomatosis with hypertrophy as the cardinal feature; it is rarely considered a cause of degenerative knee joint changes. The secondary type has been defined as lipomatosis resulting from chronic irritation of the synovium (as seen with degenerative joint disease or arthritis) rather than a true neoplasm and is by far the most common form of lipoma arborescens. Most authors have accepted the last hypothesis [14, 15, 19, 20]; this suggests that osteoarthritic changes are secondary to the presence of lipoma arborescens. Natera even concludes in his retrospective review that progressive joint degeneration could be prevented or at least delayed, if prompt synovectomy is performed [20, 21].

Patients with lipoma arborescens typically present with one or more of the following symptoms: slowly progressive swelling, pain, intermittent episodes of joint effusion, limited range of motion, and locking. Only rarely can a soft-tissue mass be identified on palpation [22]. Most patients have no history of recent trauma. The symptoms are cyclic, with intermittent exacerbations caused by mechanical trapping of the lipomatous villi inside the joint space.

The diagnosis of lipoma arborescens is based on the typical findings on MRI, but because of the clinical appearance in this case other more common pathologies had to be excluded by using laboratory tests, joint fluid aspiration, and other imaging studies. In general the laboratory findings are nonspecific. The joint fluid aspirate is negative for crystals and microorganisms and is mainly used to exclude other causes of joint swelling [2, 15].

Plain radiographs sometimes show a soft-tissue density, nonspecific bone erosion, or other osteoarthritic changes (joint space narrowing, osteophytes, subchondral sclerosis, and bone cyst formation) [6, 10].

On ultrasonography, a frondlike hyperechoic mass is seen that waves during manipulation of the joint [23].

CT scanning shows a villous synovial mass of a density similar to fat. The signal is not enhanced with intravenous contrast administration [22].

On MRI it has a pathognomonic aspect, which makes MRI the diagnostic imaging modality of choice [13]. The findings have a high degree of accuracy in the identification and anatomic characterization, and they give a correct evaluation of size and grade, which can be useful for choosing the most appropriate therapy.

These findings firstly include a large frondlike mass arising from the synovium. Secondly, the mass has a subcutaneous fat-equivalent signal on all pulse sequences. The subsynovial component has a high signal intensity on T1- and T2-weighted images and signal suppression on short T1 inversion recovery sequences (STIR) or after presaturation of the fat [10, 14, 24, 25]. There is a third characteristic: as on CT, there is no enhancement of the (sub)synovial tissue with intravenous administration of contrast medium. However, the joint fluid and synovial layer may show enhancement related to the presence of inflammatory cells [24]. A fourth feature is joint effusion, which can be present in different degrees. And finally, there is an absence of magnetic susceptibility effects from hemosiderin [26].

Other features are bone erosions, synovial cyst, degenerative changes, and chondromatosis. On MRI, our patient presented both the typical signal characteristics and a marked effusion. Furthermore, some degenerative changes such as chondropathy, osteophyte formation, and other signs of knee osteoarthritis were also seen.

The most common presentation of lipoma arborescens is a diffuse villous proliferation, but in a minority of cases it may also present as a focal pseudomass. According to Soler et al., the pseudomass type presumably is associated with primary lipoma arborescens, that is, without degenerative changes, but this was later refuted by Vilanova et al. In his retrospective series of thirty-two cases he found that 87% of the patients showed associated degenerative changes of the joint and 72% had a meniscal tear. We have to note that his study group mainly consisted of elderly patients [14, 25].

In the past, a biopsy was considered essential for making a final diagnosis, but now the typical MRI findings are sufficient to permit a reliable diagnosis [14].

Macroscopically, the lesion has a white-yellowish aspect and shows villous proliferation. Histologically, the villi are filled with mature fat cells and enlarged hyperaemic capillaries may be present. The underlying synovial membrane may contain mononuclear chronic inflammatory cells and the synovial cells may appear to be enlarged and reactive, with abundant eosinophilic cytoplasm [6, 10, 11, 25].

Lipoma arborescens forms part of the differential diagnosis of a chronic joint swelling; including pigmented villonodular synovitis, synovial osteochondromatosis, rheumatoid arthritis, intra-articular or synovial lipoma, synovial hemangioma, amyloid arthropathy, and xanthoma. Intra-articular lipoma and synovial osteochondromatosis are the only among these entities that can demonstrate similar MRI signal characteristics [25].

Intra-articular lipoma can be differentiated from lipoma arborescens based on its macroscopic and microscopic features. It appears as a solitary round or oval mass, as opposed to the multiple villous lipomatous proliferations and the frondlike morphology of lipoma arborescens. Intra-articular synovial lipoma (IASL) is composed of mature fat cells covered by a thin fibrous layer, usually with no synovial lining. Histologically, lipoma arborescens is characterized by diffuse replacement of the subsynovial layer by mature fat cells with a moderate infiltration of mononuclear cells. Intra-articular lipoma does not arise from the synovial layer, nor does it replace it.

Synovial osteochondromatosis is characterized by a nodular proliferation and metaplasia of the synovial membrane. On MRI, the signal intensity of it is similar to that of lipoma arborescens, but because of the cartilaginous nature of the lesion and the extent of the ossified or calcified regions, the signal intensity usually varies. In addition, the calcified or ossified lesions usually are already visible on radiographs [27].

Synovial hemangioma is a benign vascular malformation of the synovium which mainly occurs in children and adolescents. MRI demonstrates a lobulated intra-articular mass with a hyperintense signal due to pooling of blood in vascular spaces. Furthermore, lipoma arborescens usually arises in the suprapatellar pouch, whereas a synovial hemangioma is primarily found in the infrapatellar pad [22, 28].

The differential diagnosis of pigmented villonodular synovitis can be made by the absence of hemosiderin [28].

Joints affected with chronic rheumatoid arthritis show diffuse joint space loss, periarticular osteopenia, soft-tissue swelling, and marginal erosions on plain radiographs. Chronic rheumatoid arthritis has intermediate to low signal intensity on T1- and T2-weighted MRI-images associated with the formation of fibrous pannus. When affecting multiple joints, lipoma arborescens can mimic rheumatoid arthritis; but history, physical examination, laboratory tests, and radiography allow us to distinguish between rheumatoid arthritis and lipoma arborescens [14].

Temporary relief of lipoma arborescens symptoms may be achieved with an intra-articular injection of corticosteroids, but the recommended treatment for symptomatic lipoma is surgical resection (synovectomy) by open arthrotomy or arthroscopy [2]. Using arthroscopic techniques has the advantage that it is less invasive and it results in an early recovery. The reported outcomes of an arthroscopic resection have favorable results at six months, one year, and two years. Recurrence after synovectomy is very rare [24, 29]. The choice of one technique over the other mainly depends on the extent of involvement in the joint and on the personal experience of the surgeon.

Erselcan et al. described a case of lipoma arborescens that was successfully treated by yttrium-90 radiosynovectomy. But, there have been no additional reports of cases treated in this manner [30].

Nevertheless, when patients do not have pain or any functional limitations, a conservative treatment is indicated.

## 4. Conclusion

Lipoma arborescens is a rare intra-articular condition, characterized by villonodular proliferation of the synovium. The typical MRI appearance of fatty synovial proliferation with no other signal intensities is pathognomonic for lipoma arborescens. Although rare and benign, the condition should be considered in the differential diagnosis of a chronic joint swelling. The recommended treatment, if it causes pain and discomfort, is synovectomy. This can be performed arthroscopically or open, depending on the location and size.
